# Movement patterns and player load: insights from professional padel

**DOI:** 10.5114/biolsport.2025.139856

**Published:** 2024-07-31

**Authors:** Ricardo Miralles, Rafael Martínez-Gallego, José Guzmán, Jesús Ramón-Llin

**Affiliations:** 1 Department of Physical Education and Sport, University of Valencia, Valencia, Spain; 2 Department of Teaching of Music, Visual and Corporal Expression, University of Valencia, Valencia, Spain

**Keywords:** Accelerometer, WIMU, Sports performance, Racquet sports, Elite

## Abstract

Quantifying competition load with kinematic variables from inertial devices provides critical insights into player performance, a practice well adopted especially in team sports. This study aimed to analyse load variables in elite padel players, distinguishing between match winners and losers. Data were collected from 83 players across 23 professional circuit matches. The results highlighted specific load metrics such as distance covered, accelerations and decelerations per hour, peak speeds, and an acceleration profile per distance covered, revealing that winners exhibited significantly higher mobility than losers. Specifically, match winners travelled a greater distance per hour than match losers (Mdn_Winners_ = 2518; Mdn_Losers_ = 2339 m; p = 0.02; r = 0.25) and performed a greater number of accelerations per hour (Mdn_Winners_ = 415; Mdn_Losers_ = 382; p = 0.04; r = 0.22). These findings introduce novel data to padel, promising to refine training adjustments and offer an objective performance evaluation in both training and competitive contexts. The study’s outcomes emphasize the role of mobility in winning matches, suggesting that higher movement and acceleration rates are advantageous. This research fills a gap in padel literature, providing a foundation for future investigations into training and performance optimization strategies.

## INTRODUCTION

Monitoring player load is a common practice in high-performance sports, as it helps coaching staff understand changes in player performance, prevent possible injuries, and avoid overloads [1]. Padel studies examining external load have mainly focused on three areas: temporal structure [2], player movements [3], and game actions, including technical and tactical parameters [4]. The current study addresses temporal and movement aspects.

Regarding temporal aspects, professional point duration varies between 10 and 15 seconds, typically longer for women than for men [2]. Match duration largely depends on the number of points played and thus on the level of competitiveness between the pairs [5].

In terms of player movement, studies have primarily analysed distance covered, speed, and types of movement. Findings indicate that players cover between 2000 and 3000 m per match, half of which occurs during active play (when the ball is in play) [5, 6]. The variation in these metrics depends on the competitive level of the match, temporal aspects, or the players’ level [7, 8]. Furthermore, players spend the majority of match time at speeds below 3 km/h, and approximately 30% of the match at speeds between 3 and 6 km/h [6, 9]. This movement speed during active phases increases with the level of play [7].

The data reported by previous studies confirm that padel is an intermittent sport characterized by repeated high-intensity efforts, including accelerations, decelerations, changes of direction, and stroke execution, dispersed over a variable timeframe [10]. In other team sports, where accelerations and decelerations are considered important performance indicators, a significant number of studies have explored the specific characteristics of these types of movements [11, 12]. However, a gap in research exists regarding the study of acceleration and deceleration profiles demanded by padel, complicating the calibration of physical preparation, the implementation of injury prevention strategies, or the design of competition strategies [13].

Traditionally, the analysis of external load in racket sports was limited to quantifying the distance covered, presenting various limitations, such as not considering other aspects related to the accelerations occurring in jumps and turns [7]. Nevertheless, the use of electronic performance tracking systems (EPTS) in recent years has allowed for the analysis of other scarcely studied external load variables in padel, such as acceleration and deceleration profiles, explosive distances, and Player Load [14]. This last indicator has been reported as a reliable and valid indicator [15], showing a high correlation with physiological variables such as heart rate, $\dot{\text{V}}$O_2_max [16], and subjective scales of perceived effort [17]. For these reasons, player load has been the subject of research in a significant number of recent publications in sports such as football [18], basketball [19], and racket sports such as tennis [20].

Although previous studies have analysed performance differences in padel players based on the outcome [21], no information has been reported related to accelerations and decelerations, explosive distance, or player load. Therefore, the objectives of this study were to describe the main external load variables in professional padel players during matches, recorded using an inertial device, and to establish if there were differences between winners and losers. The equality between the winning and losing couples increases as the rounds of the draw progress, with the final being the match with the greatest equality [22]. It was hypothesised that the winners of the matches would show greater mobility than the losers.

## MATERIALS AND METHODS

### Sample

A total of 83 male professional padel players, aged between 20 and 44 years, all ranked within the 40 to 216 range of the World Padel Tour professional circuit, were recorded. In terms of laterality, 63 players were right-handed and 20 were left-handed. The analysed matches included 9 finals and 14 semifinals from the Gold Circuit 24 World Padel Tour Next Season 2021–2022. This study was performed in accordance with the ethical standards of the Helsinki Declaration. All players voluntarily participated in the study, signing an informed consent form prior to their involvement.

### Instrument

In this study, the WIMU Pro device (RealTrack Systems, Almería, Spain), regarded as a hybrid EPTS due to its GNSS and LPS technology integration, was utilized. It enables performance monitoring in outdoor locations via GNSS technology and indoor facilities where GNSS technology is limited. All data in our study were gathered from outdoor facilities. The WIMU has been demonstrated to have high validity in positioning or tracking [23].

### Procedure

In each match, data were collected from each player with their informed and signed consent, strictly adhering to the protocol established by the corresponding federation. All matches were played outdoors on glass courts. Before the general warm-ups, players were fitted with a vest without the inertial device. After completing their specific warm-up exercises and just 3 minutes before the match start, the device was placed in the vest, activated, and data recording began with the player’s first serve of the match. Data recording ended with the last point of the match. During the game, the WIMU Pro device recorded the study’s interest variables, which were also monitored in real-time using Svivo Server software (Version 2022.923.0.0) to ensure accurate data recording. After the matches, the recorded data were analysed using Spro software and stored along with other contextual variables such as match results. In compliance with privacy standards, the federation was responsible for anonymizing the data before submission to the researchers for analysis.

### Variables

This study assessed movement variables related to external load, categorized by volume and intensity criteria [7]:

Volume-related variables:

– Duration: refers to the match duration (in seconds).
– Distance: total distance covered during a match (metres).
– Relative distance: total distance covered during a match, divided by each hour of duration (m/h).

Classic volume and intensity variables:

– Maximum speed: highest speed level reached by the player (km/h).
– Acceleration +1: total number of accelerations over 1.12 m/s^2^.
– Relative acceleration: total number of accelerations equal to or greater than 1.12 m/s^2^, divided by 1 hour (n/h).
– Max acceleration: maximum acceleration reached (m/s^2^).
– Deceleration +1: total number of decelerations at -1.12 m/s^2^.
– Relative deceleration: total number of decelerations at -1.12 m/s^2^, divided by 1 hour (n/h).
– Max deceleration: maximum deceleration reached (m/s^2^).

Novel volume and intensity variables:

– Player load: Vector sum of accelerations in three orthogonal axes, used to evaluate neuromuscular loads in athletes (Cormack et al., 2013).
  $$Player\;load=\frac{\sqrt{{\left(a_{y1}-a_{y-1}\right)}^{2}+{\left(a_{x1}-a_{x-1}\right)}^{2}+{\left(a_{z1}-a_{z-1}\right)}^{2}}}{100}$$
– Explosive distance: total distance covered with acceleration greater than 1.12 m/s^2^.
– Relative explosive distance: total explosive distance in a match or training session, divided by 1 hour of duration (m/h).
– HSR relative: distance covered at speeds above the player’s threshold (default 75.5% of maximum speed), based on the player’s historical maximum speed (m).

Acceleration profile included:

– Accelerations covering distances of 1–2 m, 2–3 m, and over 3 m.
– Decelerations covering distances of 1–2 m, 2–3 m, and over 3 m.

Independent variable:

– Outcome: differentiates between the winning and losing pair of the match

### Data Analysis

For data analysis, SPSS v 28.0 (IBM; USA) and R studio (R-Tools Technology) were used. Median and interquartile range served as descriptive statistics. Initially, the Kolmogorov-Smirnov normality test was conducted. To compare load variables based on match outcomes, the Wilcoxon test was employed. Effect size was assessed using Pearson’s r, categorized as: very weak (0.0 to < 0.2), weak (0.2 to < 0.4), moderate (0.4 to < 0.6), strong (0.6 to < 0.8), and very strong (0.8 to 1.0). Significance was adjusted for p-values < 0.05.

## RESULTS

### Descriptive analysis

Table 1 displays the descriptive values of various competition load variables analysed. Notably, the median distance covered by players per match was 3430 m, with a per-hour match distance of 2401 m. The median value for players’ maximum speed was 15.21 km/h, and the Player Load was 55 arbitrary units (a.u.). The median explosive distance covered by players during matches was 399 m, translating to 253 m per match hour. The median relative HSR (high-speed running) for players was 8 m.

**TABLE 1:** Descriptives of the variables of competition load

Variable	Mean	Median (IQR)
**Volume variables**		
Duration (s)	5654.82	5116 (3345)
Distance (m)	3775.55	3439.53 (2107.78)
Distance_Rel (m)	2426.85	2407.28 (505.46)

**Volume and intensity classical variables**		
Max_Speed_(km/h)	15.32	15.20 (3.28)
Accelerations+1	615.89	587.50 (362.75)
Accelerations_Rel	394.45	395.19 (100.08)
Max_Acceleration (m/s2)	4.70	4.61 (0.98)
Decelerations+1	552.85	522.00 (332.25)
Decelerations_Rel	351.62	356.47 (109.20)
Max_Deceleration (m/s2)	-4.60	-4.54 (1.04)

**Volume and intensity novel variables**		
Player_Load_(a.u.)	63.24	55.56 (33.57)
Explosive_Distance_(m)	418.96	399.70 (243.52)
Explosive_Distance_Rel (m).	270.60	253 (118)
HSR_Rel_(m)	15.56	7.55 (13.61)

IQR = interquartile range; a.u. = arbitrary units; HSR = High Speed Running; Rel = Relative to 1 hour of play; Max = Maximum; Player Load = the vector sum of accelerations on the three axes; Explosive Distance: refers to the total distance covered with an acceleration greater than 1.12 m/s^2^.

### Analysis based on match outcome

Table 2 displays the movement variable values relative to the match outcome. Winners covered a significantly greater relative distance (median [Mdn] = 2518.25 m) compared to the losers (Mdn = 2338.55 m; p = 0.02; r = 0.25). Similarly, they performed a significantly higher number of accelerations per hour (Mdn = 415.20) than the losers (Mdn = 382.35; p = 0.04; r = 0.22). For the rest of the mentioned variables, there were no significant differences between winners and losers.

**TABLE 2:** Analysis of competition load variables based on match outcome.

Variable	Winner Mdn (IQR)	Loser Mdn (IQR)	W	p	r
**Volume variables**					
Duration (s)	5116 (3345)	4998 (3330)	918	0.74	0.04
Distance (m)	3579.16 (1987.63)	3359.94 (2113.66)	1049	0.14	0.16
Distance_Rel (m)	2518.25 (510.20)	2338.55 (427.59)	1136	**0.02**	0.25

**Volume and intensity classical variables**					
Max_Speed_(km/h)	15.12 (2.79)	15.21 (3.37)	845.5	0.75	0.04
Accelerations+1	635 (391)	560 (303)	1020.5	0.22	0.14
Accelerations_Rel	415.20 (106.10)	382.35 (79.13)	1109.5	**0.04**	0.26
Max_Acceleration (m/s^2^)	4.66 (0.94)	4.53 (1.03)	881	1	0.00
Decelerations+1	593 (371)	512 (330.5)	1008.5	0.25	0.12
Decelerations_Rel	376.46 (107.33)	326.80 (89.55)	1072	0.09	0.22
Max_Deceleration (m/s^2^)	-4.2 (1.22)	-4.58 (0.88)	932.5	0.65	0.05

**Volume and intensity novel variables**					
Player_Load_(a.u.)	57.77 (32.93)	53.24 (34.61)	1021	0.21	0.14
Explosive_Distance_(m)	400.00 (238.44)	399.41 (251.15)	1013	0.24	0.13
Explosive_Distance_Rel (m).	266.24 (116.44)	246.51 (96.56)	1015	0.13	0.17
HSR_Rel_(m)	7.26 (17.68)	8.26 (11.54)	876.5	0.98	0.01

Mdn = median; IQR = interquartile range; a.u. = arbitrary units; HSR = High Speed Running; Rel = Relative to 1 hour of play; Max = Maximum; Player Load = the vector sum of accelerations on the three axes; Explosive Distance: refers to the total distance covered with an acceleration greater than 1.12 m/s^2^.

**FIG. 1:**
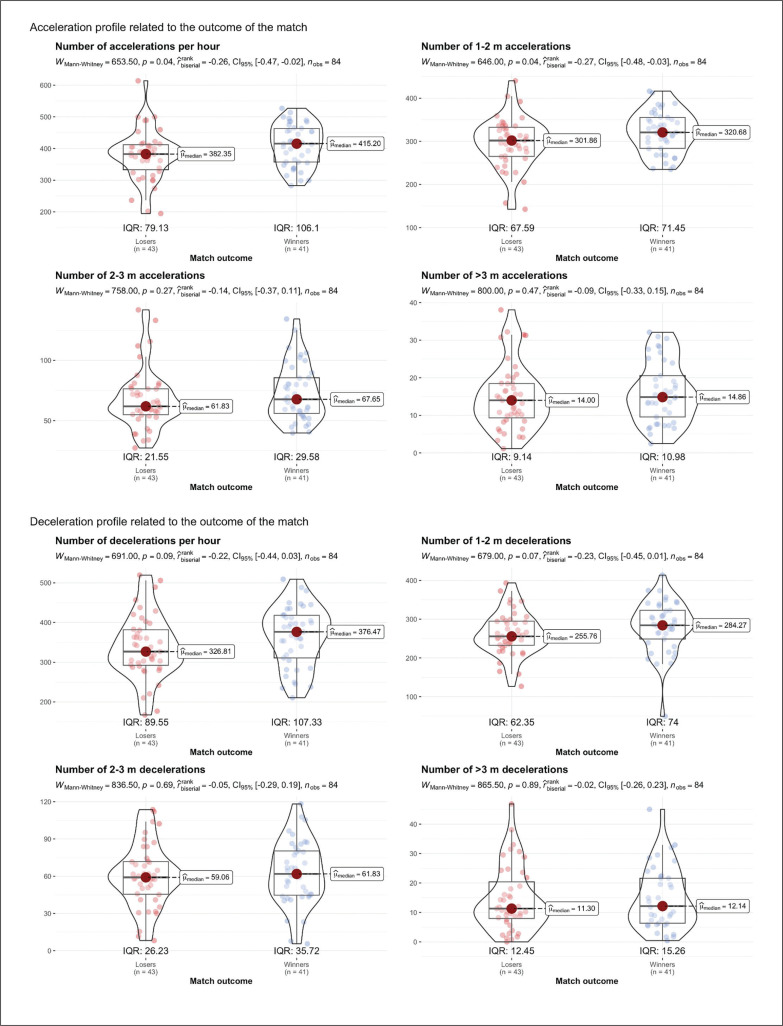
Acceleration and deceleration profile depending on the outcome of the match.

Figure 1 shows the number of accelerations and decelerations based on their distance, in relation to match outcomes. Winners performed a significantly higher number of accelerations per hour, with differences maintained in accelerations between 1 and 2 m (Mdn_winners_ = 320.68; Mdn_losers_ = 301.86; p = 0.04; r = 0.27). Notably, about 80% of accelerations occur within this distance range. Regarding decelerations, no significant differences were found between winners and losers, but winners had more decelerations over-all and across all distance ranges. Non-significant differences with moderate effect sizes were observed in total decelerations (Md-n_winners_ = 376.47; Mdn_losers_ = 326.81; p = 0.09; r = 0.22) and those from 1 to 2 m (Mdn_winners_ = 284.27; Mdn_losers_ = 255.76; p = 0.07; r = 0.23). Similar to accelerations, approximately 80% of decelerations occurred within 1 to 2 m.

## DISCUSSION

The purpose of this study was to analyse the competition load based on outcomes, using variables recorded with an inertial EPTS device related to both volume and intensity of load, as well as establishing an acceleration profile in matches of professional players.

### Volume load variables

Regarding the distance travelled, players in our study covered approximately 3440 m, while the distance per hour of play was 2407 m. These results differ significantly from those reported by Castillo-Rodríguez et al. [24] in three non-elite levels of play, which indicated lower values (between 1813 and 2319 m), and also differ greatly from the results indicated by Ramón-Llin et al. [25] in a professional circuit final where players exceeded 6000 m in two hours and 15 minutes. This may be explained by the fact that the total distance of the match depends greatly on the number of points contested, which affects the duration [26], and in our case, it was greater than that reported by Castillo-Rodríguez et al. [24] but less than that reported by Ramón-Llin et al. [25]. Additionally, in our case, the matches had an approximate duration of 85 minutes (median), which was higher than that observed [27] in professional players.

### Classic volume and intensity load variables

The maximum movement speed recorded by the players during the matches was 15.20 km/h (median values). These records are lower than those reported [25] in elite male players, who reached speeds of 25 km/h. The explanation for these differences could be as follows: maximum speeds are usually reached when the player sprints to the net [26] and even more so when a player leaves the court to return a three-metre shot from the opponent [28]. Thus, the differences in results between studies could be due to a greater presence in the sample of Ramón-Llin et al. [25] of left-handed players on the right side. We believe that right-handed players, whether in the right or left position, when smashing for three metres will force the opposing player, probably on the left side, to leave the court, so that the player on the right side will not have to leave the court for a three-metre smash when their opponents are right-handed. However, a left-handed player playing on the right side when smashing for three will probably force the opposing right side player to leave for three, and consequently, increase the maximum speed recorded by the right side player. This variation in play dynamics and player positions may also be influenced by the evolution of the game in recent years, especially considering that the data we are comparing were collected more than 10 years ago. These changes over time could further explain the discrepancies observed between the current study and earlier research.

Moreover, these sprints to the net or leaving the court on a three-metre smash explain why in our results maximum accelerations of 4.61 m/s^2^ were recorded. It is difficult to compare with other padel studies as we only found studies that established an acceleration profile for competition players, but they were in wheelchairs [29]. However, compared to sports such as football, accelerations in padel are slightly lower than those recorded by Silva et al. [30] in under-21 players who showed maximum accelerations between 4.75 and 4.89 m/s^2^, probably because the larger dimensions of the field allow greater distance to accelerate, while padel players are very limited by the dimensions of the court [31]

### Novel volume and intensity load variables

Players in our study recorded values of 55.56 a.u. in player load. We have not been able to compare these results with studies in padel, but we have with football, basketball, and tennis. In the case of football, Reche-Soto et al. [18] reported values close to 20 a.u. in third division players, while in the case of tennis, Perri et al. [20] reported between 548 and 490 a.u. In basketball, several studies [32, 33, 34, 35] have analysed player load between quarters and specific positions using WIMU Pro.

The analysis of player load has been more complex because different companies use different algorithms to classify actions and this limits comparability. [36]. Although all companies use accelerometer data from the vertical, horizontal, and medio-lateral planes to calculate player load, the calculations performed to extract the final external workload are quite different, which complicates the comparison between them [37].

### Effect of the outcome

In the analysis according to the outcome, winners covered a significantly greater distance and performed a significantly higher number of accelerations per hour than losers, with 80% of accelerations occurring over distances of 1 to 2 m. There is some controversy regarding the distance reported by previous studies, possibly due to the often close competition between winners and losers [21, 38]. In sports like padel and tennis, when losers have covered more distance than winners, it has been attributed to successful strategies of moving the opponent [7, 39]. However, in our study, we believe that the higher number of accelerations and distances covered by the winners may be due to anticipation strategies to approach the net for volleys or quickly retreat for smashes or wall returns. This argument aligns with the findings of Courel-Ibáñez et al. [40], who pointed out that winners made more winning shots from the net such as smashes or cross-court volleys, and in movements from the net backward when opponents play a lob [41]. In addition, these anticipatory strategies may be related to committing fewer unforced errors [42]. Similarly, although we have not found any studies that analyse decelerations in padel for comparison, we think that the higher number of decelerations by winners in our study could be attributed to braking in movements towards the net after the serve, following a tray shot, or after a wall return [26].

This study, while providing novel and valuable data, is not without limitations that deserve attention. The main one is the measurement of competitive intensity, based solely on playtime, without considering the score or contested points, which may not fully reflect the complexity of performance. On the other hand, the generalization of results is limited by focusing the study sample on professional players, suggesting the need to address more diverse samples in future research. In addition, uncontrolled factors such as environmental conditions and the psychological state of competitors could influence performance. Hence it would be advisable to include psychophysiological measurements and adopt qualitative approaches in future studies.

### Practical implications

There are some important implications of this study. The insights gained may help coaches, researchers, and professionals enhance training, performance, and strategy development in padel, particularly at the professional level. The study clearly implies that with professional matches, there will be a need for detailed competition load, volume, and intensity analysis, including time structure of matches/games, and design of acceleration profile. These insights will be very helpful to coaches who are preparing training programmes that simulate match activities. Coaches can, therefore, fine-tune the training to improve the performances of the players regarding fine details of the game, such as distance covered, maximum speeds accomplished, and even acceleration profiles, among many others.

## CONCLUSIONS

The analysis of internal and external load is key to adjusting and conducting more specific training sessions. In addition to distance travelled and playing time already reported by previous studies in padel, maximum speeds, accelerations, and player load are parameters recently studied in other sports to determine player performance. In the review conducted, we found no study that reported these latest data in professional padel competition. This study provided precise results of distance travelled, maximum speed, and profiles of accelerations and decelerations, which will allow coaches to determine the kinematic performance of their players in matches and training, in addition to designing specific tasks that meet the demands of competition. The comparative analysis indicated that winners exhibited greater mobility than losers.
